# Body Figure Idealization and Body Appearance Pressure in Fitness Instructors

**DOI:** 10.3389/fpsyg.2020.585901

**Published:** 2020-12-11

**Authors:** Therese Fostervold Mathisen, Jenny Aambø, Solfrid Bratland-Sanda, Christine Sundgot-Borgen, Kethe Svantorp-Tveiten, Jorunn Sundgot-Borgen

**Affiliations:** ^1^Faculty of Health and Welfare, Østfold University College, Fredrikstad, Norway; ^2^Department of Sports Medicine, Norwegian School of Sport Sciences, Oslo, Norway; ^3^Department of Sports, Physical Education and Outdoor Studies, University of South-Eastern Norway, Bø, Norway; ^4^Regional Department for Eating Disorders, Division of Mental Health and Addiction, Oslo University Hospital, Oslo, Norway

**Keywords:** group instructors, personal trainers, body image, eating disorders, drive for muscularity, drive for leanness, body figure idealization

## Abstract

**Purpose:**

The fitness centers are settings for health promotion, yet may serve as a stage for counterproductive figure idealization. Such idealization may take the form of a drive toward the thin, the muscular, or lean body figure ideal, which all hold the potential to impel an experience of body appearance pressure (BAP) and body dissatisfaction. The aim of this study was to explore figure idealization, body dissatisfaction, and experience of BAP in fitness instructors.

**Materials and Methods:**

Fitness instructors, 70 (23%) males and 236 (77%) females, were recruited through their facility chief executive officer and social media for a digital survey on mental health. Results are presented for body appreciation (BAS-2), body dissatisfaction (EDI-BD), drive for muscularity (DM), drive for leanness (DLS), questions on BAP, symptoms of eating disorders (EDE-q), and history of weight regulation and eating disorders (EDs).

**Results:**

Attempts to gain body weight were reported by 17% of females and 53% of males, whereas ∼76% of males and females, respectively, reported to have attempted weight reduction. Reasons for body weight manipulation were predominantly appearance related, and 10–20% reported disordered eating behavior. Mean BAS-2 and EDI-BD were acceptable, but 28% of females were above clinical cutoff in EDI-BD, and mean DLS were high in both sexes. In total, 8% of females were above clinical cutoff in EDE-q, which corresponded well with the self-reported ED. Approximately 90% of the sample perceived BAP to be a societal issue and reported predominantly customers and colleagues to be the cause of their personal experience of BAP. Fewer than 50% knew of any actions taken by their employer to reduce BAP. There were few differences according to profession or educational level.

**Conclusion:**

Fitness instructors report BAP to affect them negatively, which may put them at risk of impaired mental health. Educational level did not protect against figure idealization and BAP. To care for their employees and to optimize their position as a public health promoter, the fitness industry should target BAP in health promotion programs.

## Introduction

During the past 30 years, the fitness industry has expanded considerably and developed from being a small arena for body builders to large training facilities for the general adolescent and adult population (EuropeActive and Deloitte, 2020). Although being physically active is associated with many beneficial physical and psychological health effects (Garber et al., 2011; Haugen et al., 2011; Ashdown-Franks et al., 2019), several national studies find inadequate levels of physical activity in the general population (Hallal et al., 2012). Hence, there is a large potential for the fitness industry to enhance the public health by recruiting more members. A challenge with such recruitment is that non-members perceive that there is a high focus on body appearance among members of fitness centers, and as such, this prevents them from involving with this industry (Credicare, 2016). Such perceptions may be derived from the appearance-focused communication operated by the fitness industry in their marketing strategies, and by the exercise concepts consecutively introduced in the last decade (e.g., bootylicious, body pump, booty builder, 500 kcal) (Brown et al., 2017). Other than the limitation such reputation may bring for the fitness industry upon the potential to recruit new members, this also brings concern to the health of fitness instructors [i.e., group instructors (GIs) and personal trainers (PTs)] working in such environment on a daily basis. These instructors may be assumed to extend such marketing from the fitness industry, as much as they need to motivate and allure the members to choose their service or exercise sessions (Hutson, 2013). The “bodily capital” these instructors put forward may be real when members are to choose the most effective way to achieve their goal for health and body weight (BW) regulation (Hutson, 2013). Previous studies have highlighted the impact of body appearance when members are to choose their PT or GI, as this promotes credibility on health and exercise knowledge (Melton et al., 2011; Boerner et al., 2019).

Generally, when physical activity is driven by external motivation (i.e., motivation comes from outside the individual and is performed to gain a reward, e.g., admiration and popularity), and specifically when motivated by a drive for extreme body figures, it associates to less healthy, and potentially harmful, effects (Thogersen-Ntoumani and Ntoumanis, 2007; Brown et al., 2017). Within the fitness industry, idealization of extreme body figures may typically turn into a desire to attain a thin body figure (Olson et al., 1996), a muscular body figure (i.e., a desire to attain an athletic body figure characterized by large muscle mass) (McCreary and Sasse, 2000), or a lean body ideal (i.e., desire to achieve a toned, athletic body with a low level of body fat) (Smolak and Murnen, 2008). The experience of body appearance pressure (BAP) arises when the body figure idealization is reinforced with experienced expectation to comply with a certain look and hence becomes an important aspect of the self-evaluation. Importantly, BAP specifically occurs in domains or cultures where body figure associates to certain benefits and associates to several negative mental health issues (Smith Maguire, 2008; Bakken et al., 2019). The negative impact from the idealization of the traditional thin body ideals is well known (Stice and Shaw, 2002; Holland and Tiggemann, 2017), and recent findings reveal a similar impact from exposure to idealization of the lean, athletic body type (Bell et al., 2016; Robinson et al., 2017; Mathisen and Sundgot-Borgen, 2019). Also, athletes competing in sports in which there is a high focus on body appearance are at increased risk for disordered eating and eating disorders (EDs) (Bratland-Sanda and Sundgot-Borgen, 2013; Fortes et al., 2013). However, the knowledge on BAP and idealization of different body figure ideals in fitness instructors and associated risk for disordered eating is limited. Some studies show high numbers of GIs with body dissatisfaction, dieting behavior, and disordered eating (Prichard and Tiggemann, 2005; Bratland-Sanda et al., 2015). In contrast, other studies show that GIs have been found with less body dissatisfaction, assumingly because they feel confident in a fit body (Prichard and Tiggemann, 2005). None of these previous studies have investigated the association with exercise science education, which may influence the idealization of body ideals and experience of BAP. Additionally, PTs have not been included in such studies, nor have such studies included men. Furthermore, the growth and power of social media have expanded tremendously since previous publications; hence, it is timely to investigate the body figure idealization and possible consequences, in GIs and PTs of both sexes. Finally, there is rationale to believe that a high level of body appreciation (i.e., to be pleased with the way the body functions and responds to different physical challenges) may protect against the negative impact from BAP exposure and body figure idealization (Alleva et al., 2017). This effect may be specifically strong when personal experience of mastery is combined with theoretical knowledge on exercise science and health, such as those emphasized in the academic exercise science curriculum. Hence, there is a need to explore if body appreciation can protect professionals within the fitness industry against the negative impact from idealization of specific body figures, within the fitness culture.

The objective of this study was to investigate experiences of BAP, body figure idealization, and frequency of disordered eating and ED in a national representable sample of GIs and PTs of both sexes and with different educational level. We have reasons to assume no differences in experiences of BAP between GIs and PTs as both professions are found to rely on their appearance, i.e., the “bodily capital,” in order to attract customers (Melton et al., 2011; Hutson, 2013; Boerner et al., 2019). Second, following the logic from the traditional gender-related body figure idealization (Grogan, 2008), we assume male fitness instructors more typically experience high drive for muscularity, whereas female fitness instructors more typically experience a high drive for leanness (DL). As such, we hypothesized the following: (EuropeActive and Deloitte, 2020) fitness instructors of both sexes express high levels of BAP, body figure idealization, and disordered eating behavior; (Garber et al., 2011) males are more concerned about being muscular, and females are more concerned about being thin or lean; (Haugen et al., 2011) there are no differences between PTs, GIs, and those operating in combined roles (comb) in experience of BAP, body figure idealization, or disordered eating behavior; and (Ashdown-Franks et al., 2019) higher education associates to less stress about body figure idealization and appearance pressure.

## Materials and Methods

### Design

This is a cross-sectional study of PTs and group fitness instructors operating in training facilities in Norway during November 2019 and March 2020. All recruited participants responded to an electronic questionnaire, estimated to take 40 min to complete, with opportunities to pause. Participation did not include any direct benefit or remuneration.

### Participants

We contacted the chief executive officer (CEO) or the head of instructors in each fitness center registered by the Enterprise Federation of Norway “Virke Trening” or identified by official listings or by professional knowledge of the industry. These contacts were asked to distribute recruitment information by email and poster to the separate facility–CEO or directly to the fitness instructors. We also had “Virke Trening” to motivate for participation in one of their monthly newsletters. We have reasons to believe that the original and less successful recruitment strategy was partly related to poor redistribution of recruitment information. Many CEOs also reported lack of registers covering operational fitness instructors within their fitness centers and lack of direct communication channels between CEOs and individual fitness instructors. As such, after 1 month with poor response rate to recruitment, we finally recruited participants directly through social media (Facebook personal open profiles and professions group forums, and Instagram). The original intention to recruit through CEOs of fitness chains was to keep a calculation of response rate. However, because of poor estimates on numbers of operational instructors within each center, we realized such calculations could only be an estimate.

**FIGURE 1:**
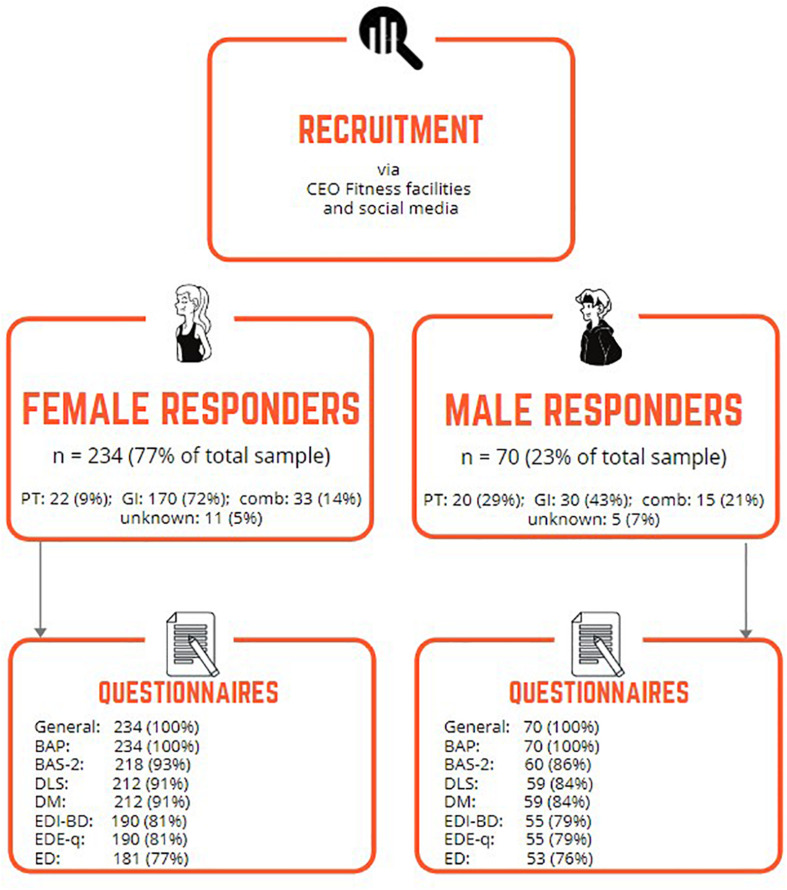
Overview of numbers (%) of responding female and male participants. CEO, chief executive officer; PT, personal trainer; GI, group instructor; comb, operating both as PT and GI; BAP, body appearance pressure; DLS, Drive for Leanness Scale; DM, drive for muscularity; BAS-2, body appreciation scale; EDI-BD, eating disorder inventory-3 body dissatisfaction subscale; EDE-q, eating disorder questionnaire; ED, eating disorders.

The recruitment invited fitness instructors to participate in a survey on motives and attitudes toward physical activity, diet, and body appearance. They were informed that results could be used to create targeted information campaigns and interventions to promote healthy work environments in the fitness industry. Respondents had to match the following inclusion criteria: operate as a PT or GI, or as a combination of both (comb), in any training facilities at the time of recruitment and to understand Norwegian written language. In total, 234 females and 70 males responded to the recruitment by fully or partially answering the questionnaire; all responses to questionnaires were included (Figure 1).

### Questionnaires

The first part of the survey asked for demographic information including age; education; profession; BW; BW history; attempts of, reason for, and methods of BW regulation; history of any ED; and experience of BAP.

#### Body Weight

Body weight history was reported as response to questions asking for the highest and lowest adult BW (i.e., after age of 18 years, and not counting any pregnancy periods). Additionally, participants were asked about attempts of any BW regulation: “Have you ever intentionally tried to reduce your body weight?” and “Have you ever intentionally tried to increase your body weight?” If responding “yes” to any of these questions, follow-up questions were presented, asking for reasons for changing their BW and which methods they had relied on in order to change their BW. All questions were presented with predefined suggestions, including a final open-ended option where participants could respond differently if needed.

#### Body Appearance Pressure

Participants responded to questions on experiences of BAP, which covered the experience of BAP in general in society, BAP at work (at fitness center, with colleagues, with customers) during leisure time, and the chance to comment on other areas of life in which BAP may occur (yes/no question, with chance to define in an open-ended response box). Additionally, participants were asked to respond to the assumed advantages of presenting a specific body figure (social recognition, followers in social media, to recruit customers, increase their chance to be employed) and how BAP affected them or their behavior (academically, socially, self-esteem, to comply with official recommendations for physical activity and diet, to follow less favorable exercise and eating routines in order to achieve the perfect body figure appearance). All questions on BAP had response alternatives on a 4-point Likert scale (1 = not at all, 4 = very much).

Participants were also asked to rate how BAP affected them, on a scale ranging from 1 (negatively) to 10 (positively). Finally, participants were asked to report if they believed colleagues experienced BAP at work, if they found their employer to prioritize BAP issues seriously [both questions had responses ranging from 1 (not at all) to 4 (very much)], and if and what concrete measures their employers had toward BAP.

### Body Appreciation Scale, Version 2

Body Appreciation Scale, version 2 (BAS-2) (Cronbach α = 0.95 in the current sample), measures body appreciation, specifically how participants are valuing their body and their level of orienting cognitive processing to protect and promote a positive view of the body (Tylka and Homan, 2015). Participants respond to 10 items on a Likert scale ranging from 1 (never) to 5 (always), with a higher average score indicating a higher level of body appreciation. The BAS-2 has been found with optimal validity and reliability in a comparable sample of Scandinavian young adults of both sexes (Lemoine et al., 2018).

### Body Dissatisfaction, Eating Disorder Inventory-3

Body Dissatisfaction, Eating Disorder Inventory-3 (EDI-3) is a 92-item questionnaire measuring symptoms of and psychometric correlates to EDs (Garner, 2004). It consists of 11 subscales, and the answers are rated on a 6-point Likert scale (always to never). The subscale “body dissatisfaction” (BD) used in this survey (Cronbach α = 0.88 in the current sample) is one of three subscales measuring risk for ED and consists of 10 items rated on a 6-point Likert scale ranging from 1 (always) to 6 (never). Scores are recoded and summarized (range, 0–40), and a total score ≥14 is identified as having a high risk for an ED (Garner, 2004). The questionnaire has not been validated for a Norwegian sample; however, evaluations in Scandinavia have found good internal consistency and validity (Kaiser and Martinussen, 2015).

### Drive for Muscularity

Drive for muscularity (DM) (Cronbach α = 0.87 in the current sample) measures any preoccupation with muscularity, having optimal reliability, and construct validity (McCreary and Sasse, 2000; McCreary et al., 2004). It constitutes 15 questions scored on a 6-point Likert scale ranging from EuropeActive and Deloitte (2020) always to Credicare (2016) never, uses reversed coding in analysis, and has a total score of 15 to 90 (high score means higher DM). The total score in the current analysis is presented as the average item score (i.e., total score from 1 to 6).

### Drive for Leanness Scale

Drive for Leanness Scale (DLS) (Cronbach α = 0.84 in the current sample) measures men’s and women’s motivating interest in having relatively low body fat and toned, physically fit muscles (Smolak and Murnen, 2008). The scale contains six items, where participants respond to a 6-point Likert scale ranging from 1 (never) to 6 (always). A higher sum score indicates greater investment in leanness. The DLS has been found valid for both men and women (Alleva et al., 2017). The total score in the current analysis is presented as the average item score (i.e., total score from 1 to 5).

### Eating Disorder Examination Questionnaire

The Eating Disorder Examination Questionnaire (EDE-q) (Cronbach α = 0.95 in the current sample) is a self-report questionnaire measuring the symptoms of EDs (Fairburn and Beglin, 2008). It consists of 28 questions, of which 22 questions are scored on a Likert scale (0–6). The 22 items are divided into four subscales (shape, BW, and eating concern, and eating restriction) and averaged into a global score, and six questions measure frequency of binge-eating and purging behavior. A cutoff global score of ≥2.5 in a Norwegian sample has previously been found to identify the probability of having an ED, whereas a global score of 1.25 (1.10) is the Norwegian normative score in a healthy cohort of comparable adults (Rø et al., 2015).

### Statistics

All data were analyzed with IBM SPSS version 26. Data were visually inspected for normality and consequently presented as mean (SD) if normally distributed or as median (range) if non-parametric.

We analyzed results for any differences between males and females, between professions (i.e., PT, GI or comb) within sexes, and between educational levels within sexes. Analyses were performed with Student independent and paired *t* test or analysis of variance for parametric data and with Mann–Whitney *U* test, Wilcoxon signed ranks test, or Kruskal–Wallis if data were non-parametric. Categorical variables were compared with *χ*^2^ test. Finally, multiple regression analyses were performed per sex to explore explanatory variables for the variability in EDI-BD and BAS-2, representing the extremes of a continuum for measure of personal relation to body figure. Variables were included in models if significantly correlated to outcomes, and variables were evaluated for independence of residuals, linearity, homoscedasticity, multicollinearity, and outliers. Significance for demographic data corresponded to *p* ≤ 0.05, but considering the explorative approach of this study, a Bonferroni correction (*p* = 0.05/103 tests) was considered too conservative; hence, *p* ≤ 0.01 was evaluated as statistically significant for all main analyses.

## Results

In total, 304 fitness instructors consented to take part in the study. Of these, 181 females (77%) and 53 males (76%) completed the whole survey (Figure 1). We found no differences between those who completed the whole survey and those that only partially responded to the questionnaire (*p* ≥ 0.12).

Most participants worked as GI (11 females and five males did not respond to question on profession) (Table 1), and mean (SD) periods of operation as fitness instructor were 10 (8.9) years among females and 9 (7.4) years among males. The majority lived and worked in cities: 194 (82%) of females and 62 (89%) of males, with the remaining reporting to work in rural areas.

**TABLE 1:** Demographic presentation of participants.

	No. (%)	Age, y	BMI, kg × m^–^^2^	PA, h/wk	Edu ≥ BSc^1^	Work h/wk
Males, total	70(23%)	37.5 (61)	25.6 (13)	5.0 (81)	16(23%)	5.0 (39)
Male PT	20(29%)	32.5 (38)	26.4 (8)	6.5 (30)	6(30%)^£^	24.0(38)^#^
Male GI	30(43%)	43(61)^#^	25.3 (13)	7.0 (30)	–	2.5(32)^#^
Male comb	15(21%)	27.0 (38)	25.0 (10)	8.0 (64)	7(47%)^£^	9.0 (31)
Females, total	236(77%)	33.0 (60)	22.7(14)*	7.0(65)*	53(23%)	4.0(70)*
Female PT	22(9%)	33 (32)	22.8 (12)	6.5 (14)	3(14%)	19.0(68)^#^
Female GI	170(76%)	34 (60)	22.7 (13)	5.0 (81)	31(18%)	3.0(42)^#^
Female comb	33(14%)	31 (37)	23.0 (9)	7.0 (21)	15(46%)^$^	10.0 (28)

Values are median (range) if not otherwise stated. NO, number; PT, personal trainer; GI, group instructor; BMI, body mass index; PA, personal physical activity; h/wk, hours per week; Edu ≥ BSc, education corresponding to, or higher than, bachelor of science; Work h/wk (at fitness center); comb, combined profession of PT and GI; ^1^Values are numbers (percent). * Significantly different to males (*p* < 0.05). ^#^Significantly different to counterparts within sex (*p* < 0.05). ^£^Significant difference between male PT and male GI (*p* < 0.001) and between male GI and male comb (*p* < 0.001). ^$^Significantly different to female counterparts (*p* < 0.001).

### BW Regulation, Lifetime, and Current Occurrence

The reported mean (SD) ideal BW deviated from actual BW by −2.6 (3.5) kg in females and −1.2 (4.5) kg in males, with significant differences between sexes (*p* = 0.005). The mean (SD) difference between highest and lowest adult lifetime BW was 12.9 (8.1) kg in females and 20.7 (11.6) kg in males, with significant differences between sexes (*p* < 0.001).

**FIGURE 2:**
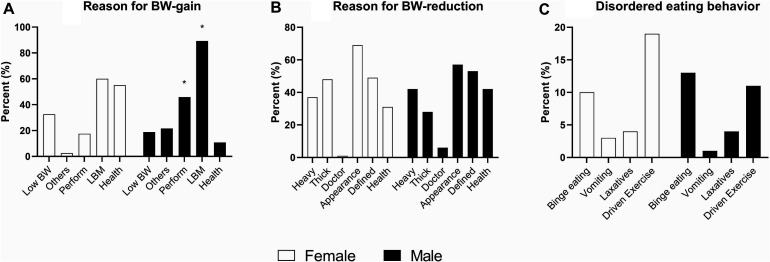
Body weight manipulations and disordered eating behavior, separately by sex. **(A,B)** illustrate the given reasons for attempts at gaining or reducing body weight (BW) (respondents could choose several reasons). Results in **(A,B)** are percent from the subsample reporting attempts for BW gain or reduction, respectively. **(C)** illustrates percent of total sample (per sexes) with current disordered eating behavior (i.e., number of episodes above the clinical cutoff). Results in **(C)** are from EDE-q items 14 and 16–18. Low BW, feeling too small; others, others have argued for the need of BW gain; Perform, to increase performance in sport; LBM, to increase lean body mass; health, to enhance health; heavy, feeling too heavy; thick, feeling too thick; doctor, doctor suggested; appearance, to look better; defined, to achieve a defined LBM look. *Significant different between sexes, *p* < 0.001.

Any attempt to gain BW was reported by 40 (17%) females and 37 (53%) males, with a significant difference between sexes (*p* < 0.001). Additionally, nine (4%) females and nine (13%) males reported to currently aim for BW gain. The median (range) age when first attempt of weight gain occurred was 24.5 (27) in females and 22.0 (25) in males (*p* = 0.42). Any attempt for weight reduction was reported by 181 (77%) females and 53 (76%) males, and 34 (14%) females and 11 (16%) males reported to currently aim for BW reduction. The median (range) age when first attempt for weight reduction occurred was 17.5 (32) in females and 23.0 (41) in males (*p* < 0.001). Females were significantly younger when they first attempted to reduce BW compared to first attempt for BW gain (*p* < 0.001), but there was no significant difference in males (*p* = 0.58). Reasons for BW gain or reduction are presented in Figure 2.

Among those reporting attempts to reduce BW, the most typical methods used by males and females, respectively, were reducing kcal intake (93 and 92%), increasing exercise volume (72 and 77%), and following self-designed diets (32 and 23%). Additionally, 14% of these females reported self-induced vomiting. Other methods (e.g., diet pills, laxatives, dehydration techniques, and popular diets) were less frequently reported (i.e., <10% of males and females).

### BAS-2, EDI-BD, DLS, and DM

The results for BAS-2, DM, DLS, and EDI-BD are presented in Table 2.

**TABLE 2:** Median (range) score in body appreciation (BAS-2), body dissatisfaction (EDI-BD), numbers (%) above EDI-BD cutoff, and mean (SD) scores in drive for muscularity (DM) and drive for leanness (DLS).

	BAS-2	EDI-BD	≥BD cutoff	DLS	DM
Males, total	4.2 (0.7)	3.0 (11.0)	4(6%)	4.2 (1.1)	2.3 (0.8)
Male PT	4.3 (0.7)	3.0 (11.0)	–	4.0 (1.2)	2.4 (0.9)
Male GI	4.1 (0.7)	3.0 (23.0)	2(7%)	4.2 (0.8)	2.2 (0.8)
Male comb	4.0 (0.6)	9.0 (21.0)	2(13%)	4.4 (1.3)	2.5 (0.8)
Females, total	3.9 (0.8)	9.0(36.0)	65(28%)	4.1 (1.0)	2.0(0.6)
Female PT	3.7 (0.8)	8.0 (29.0)	7(32%)	4.6(0.8)^$^	2.6(0.6)^#^
Female GI	3.9 (0.7)	10.0 (36.0)	47(28%)	4.1 (1.0)	1.9 (0.6)
Female comb	4.0 (0.7)	5.0 (24.0)	11(33%)	3.7 (1.0)	1.9 (0.7)

PT, personal trainer; GI, group instructor; comb, combined profession of PT and GI; BD, body dissatisfaction scale in Eating Disorder Inventory-3; DLS, Drive for Drive for Leanness Scale; DM, drive for muscularity scale; PT, personal trainer; GI, group instructor; comb, combined profession of PT and GI. * Significantly different to males (*p* < 0.003). ^#^Significantly different to its counterparts within sex (*p* < 0.001). ^$^Significantly different to female comb (*p* = 0.002).

Correlational data for body appreciation (BAS-2) are presented in Table 3. In females, 57% of the variation in BAS-2 was explained by EDI-BD (standardized β = −0.45, 99% confidence interval (CI) [−0.05, −0.02]) and EDE-q (standardized β = −0.36, 99% CI [−0.36, −0.12]) (*F*[2,187] = 124.5, *p* < 0.001) (Table 2). In males, 40% of the variation in BAS-2 was explained by EDI-BD (standardized β = −0.64, 99% CI [−0.10, −0.04]) (*F*[1,53] = 37.2, *p* < 0.001).

**TABLE 3:** Correlations between EDI-BD and explanatory variables and between BAS-2 and explanatory variables (for evaluation before multiple regression analyses).

	EDI-BD		BAS-2	
		
	*r*	*p*	*r*	*p*
**Males**				
BMI	0.15	*0.29*	−0.01	*0.94*
Age	−0.25	*0.06*	0.29	*0.03*
BW deviation	0.17	*0.22*	0.08	*0.54*
BAS-2	−0.64	***<0.001***		
EDI-BD			−0.64	***<0.001***
DLS	0.41	***0.002***	−0.15	*0.25*
DM	0.62	***<0.001***	−0.50	***<0.001***
EDE-q	0.55	***<0.001***	−0.41	***0.002***
Educational level	−0.03	*0.83*	0.09	*0.51*
**Females**				
BMI	0.40	***<0.001***	−0.19	***0.005***
Age	−0.52	*0.48*	0.06	*0.36*
BW deviation	0.58	***<0.001***	−0.45	***<0.001***
BAS-2	−0.72	***<0.001***		
EDI-BD			−0.72	***<0.001***
DLS	0.33	***<0.001***	−0.38	***<0.001***
DM	0.33	***<0.001***	−0.33	***<0.001***
EDE-q	0.73	***<0.001***	−0.70	***<0.001***
Educational level	−0.14	*0.06*	0.11	*0.09*

*BMI, body mass index; BW deviation, difference in body weight between actual body weight and subjectively reported ideal body weight; BAS-2, body appreciation scale no. 2; EDI-BD, Eating Disorder Inventory–Body Dissatisfaction; DLS, Drive for Leanness Scale; DM, drive for muscularity scale; EDE-q, Eating Disorder Examination Questionnaire. Bold numbers indicates statistical significant correlations.*

Correlational data for body dissatisfaction (EDI-BD) are presented in Table 3. In females, 67% of the variation in EDI-BD was explained by body mass index (BMI) (standardized β = 0.19, 99% CI [0.30, 1.18]), BAS-2 (standardized β = −0.38, 99% CI [−6.69, −2.87]), and EDE-q (standardized β = 0.44, 99% CI [2.42, 5.07] (*F*[3,184] = 125.9, *p* < 0.001). In males, 61% of the variation in EDI-BD was explained by BMI (standardized β = 0.12, 99% CI [−0.27, 0.84]), BAS-2 (standardized β = −0.35, 99% CI [−5.56, −0.63]), EDE-q (standardized β = 0.29, 99% CI [0.34, 4.84]), and DM (standardized β = 0.38, 99% CI [0.80, 4.37]) (*F*[4,50] = 21.8, *p* < 0.001).

### Body Appearance Pressure

Results from BAP questions are reported in Table 4. Additionally, other area of life from which the participants reported BAP was social media by 34 (14%) of females and 8 (11%) of males, and 6 (3%) of females reported self-originated BAP, women’s magazines, or dating/boyfriends as additional sources of BAP.

**TABLE 4:** Numbers (%) responding “some” or “very much” to the different BAP sources.

	SI BAP	Pers. BAP	BAP W	BAP Co.	BAP Cu.	BAP L
Males, total	63(90%)	4.2 (2.1)	13(19%)	7(10%)	13(19%)	12(17%)
Male PT	19(95%)	5.0 (7.0)	5(25%)	4(29%)	5(25%)	3(15%)
Male GI	26(87%)	4.0 (7.0)	2(7%)	–	2(7%)	5(17%)
Male comb	13(87%)	3.0 (7.0)	4(27%)	3(20%)	5(33%)	2(13%)
Females, total	224(95%)	3.6 (1.8)	37(16%)	21(9%)	37(16%)	51(22%)
Female PT	19(86%)	4.0 (7.0)	5(23%)	4(18%)	4(18%)	6(27%)
Female GI	165(97%)	4.0 (8.0)	25(15%)	13(8%)	23(14%)	39(23%)
Female comb	30(91%)	3.0 (7.0)	5(15%)	4(12%)	8(24%)	18(55%)

*BAP, body appearance pressure; SI, social issue (BAP experienced as a social issue); Pers, personal experience of BAP (ranging from 1, most negative, to 10, most positive); W, BAP at work; Co, BAP from colleagues; Cu, BAP from customers; L, BAP from leisure time.*

The majority of females 168 (71%) and males 50 (71%) believed their employer took BAP seriously. Knowledge on concrete actions taken by their employer to reduce BAP was reported by 112 (48%) females and 27 (39%) males. Among the latter, 98 (92%) females and 28 (90%) reported that these actions were well enforced.

### Eating Disorder Examination Questionnaire

Symptoms of ED evaluated by the EDE-q are presented in Table 5 and Figure 2. Other than differences highlighted in Table 2, there was a marginal difference between sexes in EDE-q global score (*p* = 0.016) and the BW concern subscale (*p* = 0.013). There were no differences between sexes in ED behavior (Figure 2).

**TABLE 5:** Median (range) EDE-q global- and subscale scores.

	EDE-q global	Above cutoff	BC	FC	EC	ER
Males, total *n* = 55	0.4 (3)	1(1%)	0.6 (4)	0.5 (4)	0.0 (2)	0.4 (4)
Male PT, *n* = 14	0.3 (3)	–	0.3 (4)	0.4 (2)	0.0 (0)	0.6 (4)
Male GI, *n* = 25	0.6 (3)	1(1%)	0.8 (4)	0.6 (4)	0.0 (2)	0.2 (4)
Male comb, *n* = 15	0.4 (2)	–	0.6 (2)	0.8 (3)	0.0 (1)	0.4 (3)
Females, total *n* = 190	0.7 (5)	21(9%)	0.8 (6)	1.1(6)*	0.2(5)*	0.6 (5)
Female PT, *n* = 20	1.1 (5)	3(14%)	1.2 (6)	1.4 (6)	0.2 (2)	1.1 (5)
Female GI, *n* = 137	0.7 (5)	16(9%)	0.8 (5)	1.0 (6)	0.2 (5)	0.6 (5)
Female comb, *n* = 33	0.7 (4)	2(6%)	0.4 (5)	0.9 (5)	0.2 (3)	0.6 (4)

Included are also numbers (%) above EDE-q global cutoff. EDE-q, Eating Disorder Examination Questionnaire; BC, body weight concern; FC, figure concern; EC, eating concern; ER, eating restriction; PT, personal trainer; GI, group instructor; comb, combined profession of PT and GI. * Significantly different to males (*p* < 0.003).

### Eating Disorders

Among those who reported to have attempted to lose BW, in total 68 (38%) of females and 5 (9%) of males believed they have had an ED (*p* < 0.001); 15 (8%) and 3 (6%), respectively, believed they currently had an ED; and 30 (17%) of females and none of the males had previously been diagnosed by a health professional.

### Effect of Education on Experiences of BAP and Figure Idealization

Separating the sample by sex and comparing those with BSc degree or greater with those having no education or separate courses with or without educational credits, we found no differences in BAS-2, DLS, DM, experience of BAP, or in personal experience of BAP (positive or negative effect). Only a marginal effect on DM in females appeared (a higher score of 0.25 in those with minor academic education, *p* = 0.014).

## Discussion

In this cross-sectional study of fitness instructors, we investigated body appreciation, body dissatisfaction (BD), figure idealization (presented as DL or DM), symptoms of EDs, and experiences with BAP and evaluated any differences in such symptoms between sexes, work profession, and educational level. Our results indicate an adequate body appreciation and generally low BD in both males and females and across professions. However, 28% of females had high presence of BD symptoms; figure and eating concern was higher in females compared to males, and DL was high in both males and females and across professions. In total, 17% of females had previously been diagnosed with an ED, and 9% of females reported current symptoms of an ED. More than 90% in the sample reported to experience BAP as a serious issue, but less than 50% were aware of concrete actions taken by their employer to reduce BAP. We found no effect from educational level on figure idealization and BAP.

### BW Regulation and Figure Idealization

In accordance with our expectation, DM was stronger among males compared to females in this sample of fitness instructors, and generally the scores echoed previous findings in general population samples (Bucchianeri et al., 2014; Sepulveda et al., 2016; de Carvalho et al., 2019). DM turned out a significant explanation to the variability of body dissatisfaction in males only, and significantly more males compared to females aimed for an increase in BW, with reasons mainly related to a wish for increased lean body mass and performance or health. These findings highlight a gender-specific body figure idealization, and while females traditionally have attained much attention for issues related to BAP and specifically the desire for thinness (Stice and Shaw, 2002; Grogan, 2008), this finding highlights the need to use suitable questionnaires to capture the body figure issues in males and accordingly targeted interventions to reduce the impact of such figure idealization and BAP.

While some of our findings support our hypothesis on females being more concerned about a slim figure appearance compared to males (e.g., a higher score in BW-, body figure-, and eating concern, respectively), there were no sex differences in DL. Approximately three-fourths in both genders reported to have attempted to lose BW, with reasons mainly related to appearance and less related to health and functionality. Hence, fitness instructors of both sexes seem to idealize an athletic body figure; still, males aim for a more bulky, lean athletic appearance, whereas females aim for a more toned, lean athletic appearance. Concerning the females, the high DL, a high number with BD, and the high frequency of disordered eating behavior bring concern for their health. Compared to previous reports, these current findings indicate an increase in such issues in the past 5–18 years in female fitness instructors (Höglund and Normén, 2002; Bratland-Sanda et al., 2015). The high frequency on self-reported history of EDs and the numbers of participants with current symptoms of EDs underline the severity of being exposed to such lean, athletic figure idealization.

### Body Appearance Pressure

Both males and females reported BAP to be a real societal issue, and whereas males seemed to be neutral on how BAP impacted their well-being, females in general rated it as negative. Importantly though, discussing body image issues does not reflect typical masculine traits and has been described by young men as “a girl thing” (Hargreaves and Tiggemann, 2006). Such attitudes might bias male’s ability to report high levels of BAP, and the high numbers of males reporting attempts in weight gain or reduction, and higher DM compared to females, may imply some issues possibly originating from BAP. Hence, while minimizing the level of reported BD and how BAP affects them, the DL and DM, concurrently to the reported BW changes, reveal investment in body appearance interests. Being a PT seemed to be more frequently associated with figure idealization and experiences of BAP, as GIs less frequently reported such experiences compared to PTs and those with combined operation. These results echo previous findings on the experienced “body capital” a PT, in particular, must display in order to recruit new customers (Melton et al., 2011; Hutson, 2013; Boerner et al., 2019) and may relate to the fact that GIs more likely attract customers based on the atmosphere they create in the group setting and experiences of social belongingness.

Summarizing our findings on body figure idealization and BAP, fitness instructors as a professional group are in general coping adequately. This may be the result from their personal, regular exercise regimens, resulting in a body appearance and functionality satisfying their personal expectations (Alleva et al., 2017). Being body appreciative is considered to have a protective effect on BAP and results in flexibility to any negative body experiences, such as body dissatisfaction (Rogers et al., 2018). Still, the reported disordered eating behavior, BW regulation attempts, and DL reveal a continuous striving to maintain their appearance or also to adjust to the more extreme, defined athletic look. Such strivings are highlighted by the high number reporting the experience of BAP.

The majority of fitness instructors believed their employer to take BAP seriously; still, about 30% reported no such priorities within their fitness center. Importantly, fewer than half of the fitness instructors were aware of any concrete actions taken by their fitness center to reduce BAP. As such, an important responsibility lies within the fitness industry to improve their creation of, and communication about, stances toward BAP and actions applied. To create a more body image–positive exercise context for both customers and employees, changes within the marketing campaign of exercise, such as the presentation of reasons for exercising, the marketing of the PTs, and the communication within the center and between the employees need to reflect appearance focus in a lesser extent. Hence, to avoid further reinforcement of today’s massive body appearance focus, the fitness industry should promote a contrasting body functionality focus and communicated with a broader body appearance diversity. Although this has been in the spotlight in the last decade, there is still work to be done (Fjellanger, 2019; Johansen and Røssland, 2019).

### Profession and Educational Level

There are good reasons to believe health-related education such as BSc or MSc programs in exercise science increases skepticism against figure idealization and awareness of and embracement on the many beneficial health effects from physical activity (Malek et al., 2002). However, as long as there is a societal body idealization of a lean and toned body, it is hard not to be influenced by that culture (Håman et al., 2017). Additionally, with the strong association of BMI and health (Guh et al., 2009; Bhaskaran et al., 2018) and expectations to match a certain physique as a way of increasing one’s credibility as a health professional (Melton et al., 2011; Hutson, 2013; Boerner et al., 2019), figure idealization and BD may be just as true for those with higher education. Furthermore, if education does not include body image topics within their curriculum, such as reflecting upon body talk and body communication, self-representation, promotion of body appreciation, and reduction of dissatisfaction risk factors within an exercise environment, improvements may not be expected. Just as important is increased (mass and social) media literacy to reduce the risk for idealization of unhealthy ideals in fitness instructors (McLean et al., 2016) and to relearn evidence-based exercise and nutrition information, which might help them resist the unhealthy information promoted in social media by influencers (Pilgrim and Bohnet-Joschko, 2019). Knowledge on EDs and dysfunctional exercise (e.g., excessive exercise, compulsive exercise) is unfortunately low in primary health care, among health professionals, and in the fitness industry specifically (Mond et al., 2010; Kornstein, 2017; Yager et al., 2017; Bullivant et al., 2020). As much as formal education in sports and exercise relates to knowledge on EDs (Bratland-Sanda and Sundgot-Borgen, 2015), currently, neither health profession educations nor course programs in PT do sufficiently address these topics. Hence, there is a need to increase ED mental health literacy within the fitness industry and to incorporate action plans in order to support efficient and safe actions.

### Strengths and Limitations

Strengths of this study are the use of validated questionnaires, measuring symptoms of EDs, and a broader perspective of figure idealization than previously reported. This study is also the first to measure BAP and experienced effects from BAP. Additionally, this study included both sexes and PTs and GIs and evaluated the effect of educational level. Limitation of this study pertains to the lack of validation of the questionnaires EDI-BD, DM, and DLS in Norwegian samples; however, reliability in the current sample was high. Further, the authors’ self-developed questions and items lack validation, and we acknowledge that this limits those findings. Limiting our findings is a weakness in our questionnaire coding, leaving only those reporting attempts of weight reduction to self-report their history or presence of EDs. Concerning the latter, the majority reported attempts on weight loss (>76%), and frequency of self-reported ED perfectly matched the identified high-risk cases by use of EDE-q. As such, it is reasonable to suggest our numbers reflect a true prevalence of EDs in this recruited sample. Further, the use of cross-sectional design precludes us from suggesting any cause–effect, and measuring BAP with unvalidated questions may bring concern for the validity of the findings. Also, the lack of a national or center-specific overview of numbers of employees working as fitness instructors makes it difficult to suggest any response rate and as such may put our results in doubt with regard to its generalizability. Finally, a suggestion for future, prospective studies would be to include clinical interviews, at least for measures that may be used for diagnostic purposes (such as EDs or body dysmorphia).

Working in the fitness industry, which appears to be a highly appearance-focused industry, may impair health. We did not find higher education to protect against figure idealization and BAP. To care for their employees and to improve their position as a public health promoter, the fitness industry should implement BAP prevention strategies and change their marketing strategy. Educational program courses addressing body acceptance, reflections on BD, improved knowledge on exercise for health, nutrition for health and performance, mental health literacy, and social and mass media literacy seem to be needed.

## Data Availability Statement

The raw data supporting the conclusions of this article will be made available by the authors, without undue reservation.

## Ethics Statement

The studies involving human participants were reviewed and approved by the Norwegian Regional Committees for Medical and Health Research Ethics (No. 28855). The Norwegian Centre for Research Data (No. 868768). Registered in the Clinical Trial Registry (No. NCT04135729). The patients/participants provided their written informed consent to participate in this study.

## Author Contributions

TM recruited participants, kept communication with fitness centers, did the statistical analyses, wrote the draft for the manuscript, and finished the document before submission. JA created and kept logistics of online questionnaires, recruited participants, communicated with fitness centers, controlled the statistics, and reviewed the final manuscript. SB-S controlled the statistical analyses, wrote the draft for the manuscript, and reviewed the final manuscript. CS-B, KS-T, and JS-B wrote the draft for the manuscript and reviewed the final manuscript. All authors contributed in the planning of this study.

## Conflict of Interest

The authors declare that the research was conducted in the absence of any commercial or financial relationships that could be construed as a potential conflict of interest.
